# Effect of Cellulase Enzyme Treatment on Cyst Wall Degradation of* Acanthamoeba sp*.

**DOI:** 10.1155/2019/8915314

**Published:** 2019-03-28

**Authors:** Tisha Lazuana, Hendri Astuty, Ika Puspa Sari

**Affiliations:** ^1^Master Program in Biomedical Sciences, Faculty of Medicine Universitas Indonesia, Indonesia; ^2^Department of Parasitology, Faculty of Medicine Universitas Indonesia, Indonesia

## Abstract

**Aim:**

The goal of this study is to know the potential of cellulase in the degradation of cyst wall* Acanthamoeba sp*.

**Methods:**

Sample of* Acanthamoeba sp*. obtained from isolate collection of Department of Parasitology FKUI of which two samples come from patient and one sample is from environment. All three samples were cultured using non-nutrient agar (NNA) media and identified by PCR and sequencing. The concentration of cellulase concentration used was 50 U, 100 U, 150 U, 200 U, 250 U, and 300 U with the incubation time used being 2 hours, 4 hours, 6 hours, 8 hours, and 24 hours. Furthermore, treatment results with the most optimum concentration and incubation time were observed by using SEM to see changes in the surface of the walls of the cyst. A cysticidal test was performed to determine the effectiveness cysticidal action of disinfectant solution, cellulase, and the combination of disinfectant solution and cellulase in killing* Acanthamoeba sp*. cyst assessed by their viability value.

**Results:**

The most optimal cellulase concentration in killing* Acanthamoeba sp.* cysts was 300 U with an incubation time of 24 hours. Percentage of viability of* Acanthamoeba sp*. which was exposed to a disinfectant solution for 24 hours was 95%, cellulase alone for 24 hours 75%, and the combination of cellulase and disinfectant solution for 24 hours 25%.

**Conclusions:**

Cellulase is capable of degrading* Acanthamoeba sp*. cyst wall. Optimal cellulase concentration in degrading* Acanthamoeba sp*. cyst wall is 300 U with an optimal incubation time being 24 hours. The addition of cellulase to the disinfectant solution has the potential to increase the effectiveness of the disinfectant solution because cellulase is capable of degrading the cyst wall allowing the disinfectant solution to enter and kill* Acanthamoeba sp.* cysts.

## 1. Introduction

*Acanthamoeba sp.* is one of free-living amoeba (FLA), which is an opportunistic pathogen widely spread in the environment [1]. According to 18s rRNA sequence,* Acanthamoeba sp.* genus can be divided into 20 genotypes, T1-T20 [2]. Recent studies showed that* Acanthamoeba* T4 genotypes are the main cause of* Acanthamoeba* keratitis (AK), a severe progressive corneal inflammation which can lead to blindness [3].

*Acanthamoeba* keratitis is often associated with contact lenses misuse [4]. According to the survey, complications towards contact lenses use have arisen with the increase of contact lenses user over time; around 4-10% of contact lens users underwent mild irritation to blindness [5]. Each year around 80.000 people suffer from eye disease due to contact lens use [6]. Contact lens users have increased by around 15% each year in Indonesia. However the keratitis occurrence was rarely reported, because of undistinguishable clinical manifestation between fungal and bacterial infection [7].

Studies were performed to evaluate the effectiveness of contact lens disinfectant solution on eliminating contaminant from bacteria or parasites. Siddiqui R et al. (2014) have shown that none out of 9 disinfectant solutions tested was effective in killing* Acanthamoeba sp.* cysts [8]. Similar result was brought by Padzik M et al. in the same year in Poland: none out of 4 disinfectant solutions tested has amoebicidal effect towards* Acanthamoeba sp.* [9]. In addition, Abjani F et al. (2016) discover that chlorhexidine alone or mixed with disinfectant solution was still ineffective in eliminating* Acanthamoeba sp. *cysts [10].

*Acanthamoeba sp.* cyst wall consists of ectocyst (laminar, fibrous outer layer) and endocyst (fibrillar layer) [11]. Ectocyst itself contains protein and complex polysaccharides, while endocyst contains cellulose, a polymer of glucose with *β*1*⟶*4 glycosidic bonds. Cellulase is an enzyme that can damage this glycosidic bond, which can be the target in eliminating* Acanthamoeba sp.* cyst [12].

Cellulose degradation is a potential therapy in targeting trophozoite wrapped by cyst wall effectively. Thus cellulase can damage the* Acanthamoeba* cyst wall [13]. The morphological change between normal cyst and cellulase treated cyst was observed with a fluorescent microscope and Scanning Electron Microscopy (SEM) method.

## 2. Method

Three samples used in this study were obtained from 2 patients* Acanthamoeba* keratitis (AK) and 1 sample from the environmental water in Department of Parasitology, Faculty of Medicine, University of Indonesia.

### 2.1. Culture and Identification

*Acanthamoeba sp. *from Department of Parasitology collection were regrown in pages-salt agar media and stored at 30°C for 14 days to form a cyst. Total cysts formed were counted with hemocytometer [10].* Acanthamoeba sp.* DNA extraction was performed with Qiagen kit, followed by Polymerase Chain Reaction (PCR) targeted to 18s rRNA with primers JDP1 (5'GGCCCAGATCGTTTACCGTGAA-3') and JDP2 (5'TCTCACAAGCTGCTAGGGGAGTCA-3') to yield a 423-551 bp specific amplicon [14].

### 2.2. Cellulase Concentration Optimization

The cellulase used is commercial cellulase obtained from MP Biomedicals (Santa Ana, USA) and originating from* Aspergillus niger*. One unit (U) will liberate 1 *μ*m of glucose from cellulose in one hour at pH 5. Cellulase enzyme preparation was carried out by dissolving the cellulase enzyme in acetate buffer with a pH of 4.5-5.5.

Six tubes were filled with 100*μ*L of the sample with 5x10^3^ of total cysts in each tube. To each tube, cellulase was added with serial concentration as follows: 50U, 100U, 150U, 200U, 250U, and 300U, and then incubated in room temperature for 24h. Trypan blue 0.1% was added to the cysts for quantification with a hemocytometer.

### 2.3. Cellulase Incubation Time Optimization

Five tubes were prepared with 100*μ*L of a sample containing 5x10^3^ of total cysts in each tube. Cellulase was added to each tube with the optimum concentration, followed by incubation for 2h, 4h, 6h, and 24h, respectively, to each tube. Remaining cysts were counted with hemocytometer Trypan blue in 0.1%.

### 2.4. *Acanthamoeba sp.* Cyst Morphological Examination with SEM

In this study, Scanning Electron Microscope used was JSM-6510LA. Morphological examination is done by dividing* Acanthamoeba sp.* into 2 tubes; one contains 300 U of cellulase. Samples were incubated for 24h, followed by morphological examination by SEM. To observe the sample with SEM, the sample was fixed with glutaraldehyde 2% in 0.1 M cacodylate buffer with pH between 6.8 and 7.4. The sample was centrifuged at 500 g for 2 minutes. Then the pellets were fixed again with 2% osmium tetroxide, dehydrated with ethanol and propylene oxide, filtered with a millipore filter (diameter 22 mm), dried for 24 hours, then stained with contrast uranyl acetate and citrate, and then observed under SEM.

### 2.5. Cellulase, Disinfectant Solution, and a Mixture of Cellulase and Disinfectant Solution Effectivity Assay

Effectivity assay was assessed to compare the cysticidal activity of cellulase, disinfectant solution, and a mixture of both in discarding* Acanthamoeba sp.* cyst. Three tubes were prepared with 100*μ*L of a sample containing 5x10^3^ of total cysts. 100*μ*L of cellulase 300U was added to the first and third tube, while 100*μ*L of disinfectant solution was added to the second and third tube. Each tube was then incubated in room temperature for 24h. Surviving cysts were counted with hemocytometer in 0.1% Trypan blue. Acridine orange staining was also performed to evaluate the different cyst wall morphology among treatments.

## 3. Results

### 3.1. Cellulase Concentration and Incubation Time Optimization

**Figure 1:**
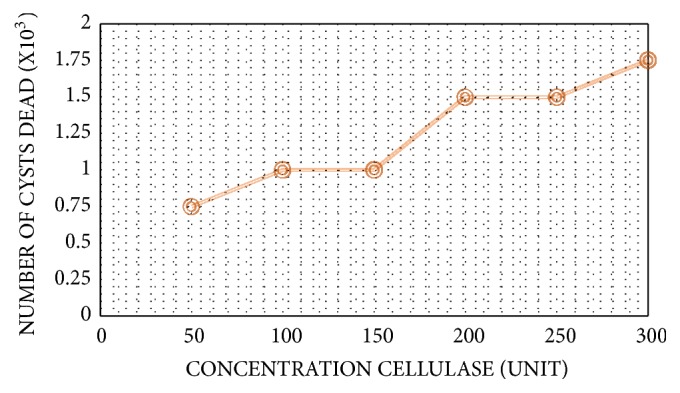
Cellulase concentration optimization.

**Figure 2:**
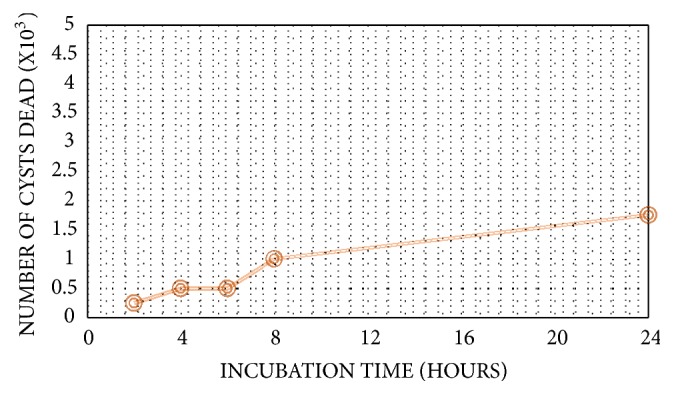
Incubation time optimization.

Both samples from AK patients were identified as* Acanthamoeba castellani* T4 genotype, and sample from biosphere was identified as* Acanthamoeba lenticulata* T5 genotype. According to cellulase optimization step,* Acanthamoeba castellani* T4 was found to be the most pathogenic genotype. Optimal cellulase concentration to discard* Acanthamoeba sp.* was 300U with 24h of incubation time (Figures 1 and 2).

**Figure 3:**
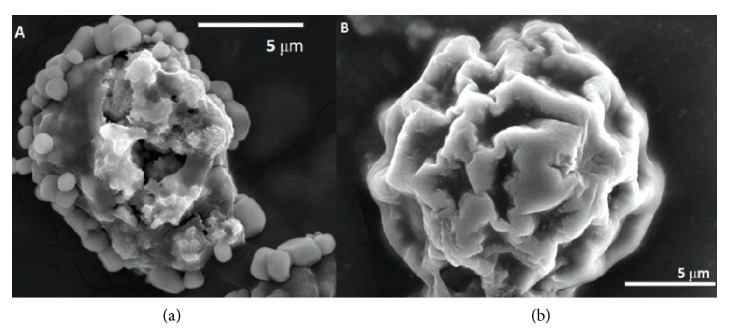
A control sample (a) and treated sample with cellulase enzyme 300 U (b) after 24 hours incubation morphological structure in SEM examination.

*Acanthamoeba sp. cyst morphological examination using SEM.* Different morphological structure in cyst surface between control and 300U, 24h cellulase treated was shown in Figure 3. On the control sample, cyst surface was found to be granulated, while on the treated sample the cyst surface was discovered to be smooth and impaired.

**Figure 4:**
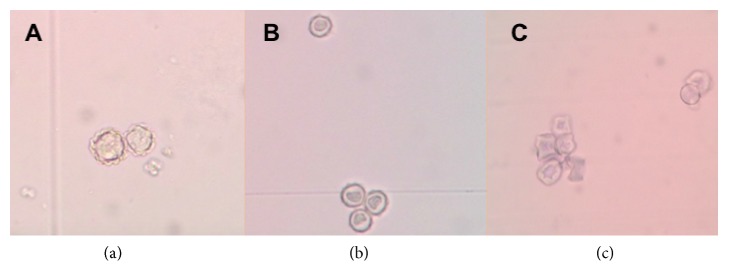
Microscopic structure of* Acanthamoeba* cysts (40x10 magnification) after 24h incubation with a disinfectant solution (a), cellulase enzyme 300 U (b), and a mixture of cellulase 300 U and disinfectant solution (c).

*Result of cellulase, disinfectant solution, and a mixture of cellulase and disinfectant solution effectivity assay*. Killing effectivity was stipulated based on* Acanthamoeba sp*. viability after treatment. Disinfectant solution treatment showed intact cyst, with double layer cyst wall and perfectly round shape of cyst after 24h treatment. Cellulase treatment for 24h caused cysts shrinkage and dwindled cytoplasm. However cyst exterior still looked round. A mixture of cellulase and disinfectant solution 24h treatment lead to cysts shrinkage, reduced cytoplasm volume, and irregular wrinkled cysts (Figure 4).

**Figure 5:**
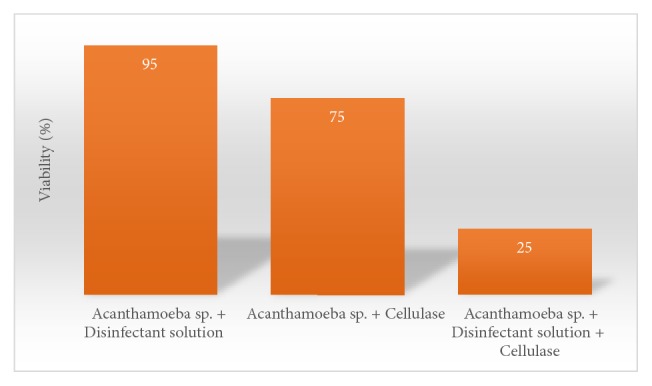
The viability of* Acanthamoeba sp.* after 24h incubation in a disinfectant solution, cellulase enzyme (300U), and a mixture of disinfectant solution and cellulase 300 U.

*Acanthamoeba* sp. viability after 24h incubation with disinfectant solution, cellulase 300U, and a mixture of both is 95%, 75%, and 25%, respectively (Figure 5). Cellulase 300U addition to disinfectant solution was discovered to reduce the* Acanthamoeba sp.* cyst viability to 25%.

**Figure 6:**
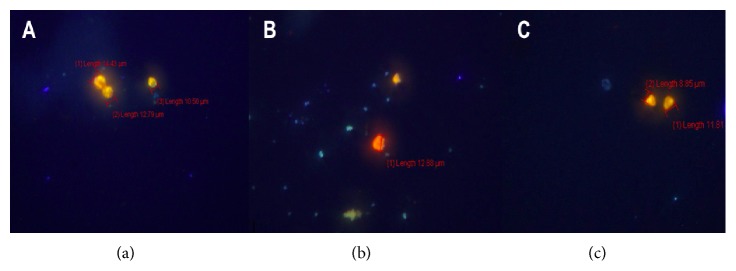
Fluorescent Microscopic examination with acridine orange staining. Acanthamoeba sp. Incubated with disinfectant solution (a), cellulase 300 U (b), and a mixture of disinfectant solution and cellulase 300 U (c).

There was a morphological difference on cyst wall after treatment. Disinfectant solution treatment for 24h showed that the cyst still has its round shape and intact with normal cyst size (10-14*μ*m) (Figure 6(a)). Cellulase 300U 24h treatment resulted in smaller cyst size (12*μ*m) with imperfect round shape (Figure 6(b)). Meanwhile, mixture of cellulase 300U and disinfectant solution resulted in shrunk (8-11*μ*m) and irregular cyst shape (Figure 6(c)).

## 4. Discussion

*Acanthamoeba* keratitis (AK) is a corneal infection caused by a microscopical organism, a Free-Living Amoeba (FLA) known as Acanthamoeba, and normally found in the waterbody (lake, ocean, and river) as well as tap water, swimming pool, tub, soil, and air.* Acanthamoeba* has 20 genotypes identified so far which are T1-T20 [15]. T4 genotype is the most common and dominant among all genotypes. In addition, T4 genotype was reported as the most pathogenic genotype and most common to be found in AK disease among other genotypes [16].

In this study, we identified 2 samples from AK patients and 1 sample from the environment. All DNA samples were amplified with PCR technique, yielded a ~450 bp amplicon length, followed by gene sequencing to reveal its genotype. Sequencing result from B, W, and S samples showed 432bp, 433bp, and 424bp amplicon length, respectively. These sequences were compared to* Acanthamoeba* sp. sequence published in* GeneBank* to calculate the percentage of similarity through NCBI BLAST.

B and W samples which came from AK patients have 100% sequence similarity to* Acanthamoeba castellani *strain T4, and S sample obtained from the environment has 100% sequence similarity to* Acanthamoeba lenticulata* strain T5. This result is similar to the previous study which stated that* Acanthamoeba* T4 was the main cause of AK [17]. Conversely, it is not simply said that* Acanthamoeba* lenticulate T5 was incapable of causing AK. Spanakos G et al. in 2006 discovered the AK cases with* Acanthamoeba* lenticulata T5 as the main cause of this disease [18].

AK disease is often related to the contact lens utilization. Incorrect usage and upkeep become the main risk factor of AK [19]. Previous studies have shown that disinfectant solution with PHMB active ingredients was not effective in eliminating* Acanthamoeba*, especially in cyst stadium.* Acanthamoeba* cyst has 2 layers of cyst walls which is difficult for the disinfectant solution to penetrate [8]. Cellulose is the most abundant component in* Acanthamoeba* cyst wall. Cellulase addition has a high chance in degrading* Acanthamoeba* cyst wall and hence will allow the disinfectant solution to penetrate to the cyst [13].

In this research, optimum cellulase concentration to degrade* Acanthamoeba* cyst wall was gained by optimization. The sample used in this step was B sample, which was the* Acanthamoeba* T4 genotype, due to its pathogenicity, and the most common cause of AK. B sample was chosen over W sample because B sample contains more cysts than W sample. Total cyst used for cellulase concentration and incubation time optimization was 5 x 10^3^.

Series of concentration tested for optimization was 50U to 300U, incubated for 24h, with 5 x 10^3^ of total cyst in 100 *μ*L. Results of concentration optimization are as follows: 0,75 x 10^3^, 1 x 10^3^, 1 x 10^3^, 1,5 x 10^3^, 1,5 x 10^3^, and 1,75 x 10^3^ cysts were eliminated in 50U, 100U, 150U, 200U, 250U, and 300U, respectively. Other than cellulase concentration, the incubation time was also determined in this research. Series of incubation time as follows: 2h, 4h, 6h, 8h, and 24h, was performed to discover the optimum incubation time. 24h was found to be the optimum incubation time as it eliminated 1,75 x 10^3^ of the cysts. These combined results showed that cellulase alone (300U) for 24h was still ineffective in banishing* Acanthamoeba* cyst. Only 1,75 x 10^3^ cysts were eliminated, out of 5 x 10^3^ of the total cysts.

Next step was to observe the* Acanthamoeba* morphological change after cellulase treatment. Untreated and 24h cellulase 300U-treated* Acanthamoeba* were noted and compared. SEM result revealed that untreated* Acanthamoeba* has granulated surface while cellulase-treated* Acanthamoeba* has smooth, degranulated, and impaired surface.

A further step was to determine the cysticidal capability of cellulase and disinfectant solution. Samples were incubated with cellulase, disinfectant solution, and a mixture of both for 24h. Results showed that 95%, 75%, and 25% of cyst were viable after incubation with disinfectant solution, cellulase, and a mixture of both, respectively. It was clear that a mixture of cellulase and disinfectant solution is more effective in eliminating* Acanthamoeba* cyst until 25% of viability.

Cyst morphology after cellulase; disinfectant solution; and a mixture of both cellulase and disinfectant solution-treatment was observed. Disinfectant solution treatment for 24h showed that the cyst morphology was similar to normal cyst: 10-14*μ*m size and a round shape. Cellulase 300U for 24h treatment did reduce cyst size to 12*μ*m and yield to irregular cyst shape, while a mixture of cellulase 300U and disinfectant solution treatment for 24h caused the cyst size reduction to 8-11*μ*m and irregular cyst shape.

*Acanthamoeba* cyst was more susceptible to antiamoeba compared to trophozoite, due to a double layer of cyst wall in* Acanthamoeba*, mainly consisting of cellulose [20]. Hence, inhibition in cellulose synthesis may be potential in preventing* Acanthamoeba *encystation. In other words, cellulase may rise as a useful additional treatment in eliminating* Acanthamoeba* [21].

Cellulose was synthesized in certain organisms, including vascular plant, major algae, fungi, and certain bacterial species. Mammal, on the other hand, does not synthesize cellulose, thus bringing cellulase as a suitable antimicrobial candidate. Cellulose 2,6-bichlorobenzonitrile synthesis inhibitor was mentioned to be able to prevent* Acanthamoeba* encystation [22]. Targeting precursor in cellulose biosynthesis was also proven to be useful.* Acanthamoeba* stored glycogen as the building block of cellulose synthesis. Previous studies have shown that glycogen was drained along the encystation process, resulting in interference of glycogen phosphorylase mRNA expression and eventually blocking encystation process [23].

Neither disinfectant solution nor cellulase alone was effective in eliminating* Acanthamoeba*, as disinfectant solution alone could not penetrate the double cyst wall, and cellulase alone is not capable of inhibiting encystation but still depends on amoebicidal activity to banish* Acanthamoeba*. Cellulase and disinfectant solution together allowed the cellulase to degrade the cyst wall and disinfectant solution to target the trophozoite cell membrane. Overall,* Acanthamoeba* trophozoite is extremely sensitive to the disinfectant solution, as well as a mixture of cellulose and disinfectant, which has proven to eliminate both trophozoite and cysts. Although we still have to discover the toxicity effect of this new formulation on the host; however, this finding showed that the addition of cyst wall degrading molecule in disinfectant solution would increase the* Acanthamoeba* elimination effectivity [10].

This research is limited but unable to observe the morphological change on* Acanthamoeba sp.* cell component after treatment, due to inadequate equipment, where SEM was only able to observe the cell wall surface. Thus, this research was unable to further analyze the potency of cellulase and the effectivity of cellulase addition to the* Acanthamoeba sp.* disinfectant solution.

## Data Availability

The data (study result such as figures) used to support the findings of this study are included in the article. Additional data are available from the corresponding author upon request.
